# Factors Determining the Increased Risk of Falls in Individuals With Knee Pain in the Malaysian Elders Longitudinal Research (MELoR) Study

**DOI:** 10.3389/fmed.2019.00277

**Published:** 2019-12-03

**Authors:** Sumaiyah Mat, Azad Hassan Razack, Jasmine Lim, Su-Yen Khong, Shahrul Bahyah Kamaruzzaman, Ai-Vyrn Chin, Azlina Amir Abbas, Noran Naqiah Hairi, Sajaratulnisah Othman, Maw Pin Tan

**Affiliations:** ^1^Ageing and Age-Associated Disorders Research Group, Faculty of Medicine, University of Malaya, Kuala Lumpur, Malaysia; ^2^Geriatric Division, Department of Medicine, Faculty of Medicine, University of Malaya, Kuala Lumpur, Malaysia; ^3^Department of Surgery, Faculty of Medicine, University of Malaya, Kuala Lumpur, Malaysia; ^4^Department of Obstetrics and Gynaecology, Faculty Of Medicine, Kuala Lumpur, Malaysia; ^5^Department of Orthopaedic Surgery, Faculty of Medicine, National Orthopaedic Centre of Excellence in Research and Learning (NOCERAL), University of Malaya, Kuala Lumpur, Malaysia; ^6^Department of Social and Preventive Medicine, Faculty of Medicine, Centre for Epidemiology and Evidence-Based Practice, University of Malaya, Kuala Lumpur, Malaysia; ^7^Department of Primary Care Medicine, Faculty of Medicine, University of Malaya, Kuala Lumpur, Malaysia; ^8^Centre for Innovation in Medical Engineering, University of Malaya, Kuala Lumpur, Malaysia; ^9^Department of Medical Sciences, School of Healthcare and Medical Sciences, Sunway University, Bandar Sunway, Malaysia

**Keywords:** accidental falls, aged, osteoarthritis, disability, depression

## Abstract

**Objectives:** While the negative impact of falls in older persons has been recognized, the association between knee pains and falls remains inconclusive due to underreporting and undertreatment of knee pain. This study was conducted to evaluate the relationship between knee pain and knee pain severity with falls risk and to further determine factors which influence this potential relationship.

**Design:** This was cross-sectional study from the Malaysian Elders Longitudinal Research (MELoR) study.

**Setting:** Urban community dwellers in a middle-income South East Asian country.

**Participants:** One thousand two hundred twelve of a representative sample of community dwelling older persons aged 55 years and older.

**Outcome measures:** Falls in the preceding 12 months and knee pain were collected during a home-based computer-assisted interview. Physical and functional performance were measured using the Timed Up and Go test and the Katz and Lawton scales, respectively. Psychological status was determined using the Depression Anxiety and Stress Scale (DASS-21).

**Results:** Of the 1,212 participants included in this analysis, knee pain was present in 402 (33.17%) individuals (124 (30.85%) mild, 210 (52.24%) moderate, 68 (16.92%) severe). The presence of knee pain was associated with increased risk of falls [odds Ratio, OR(95% confidence interval, CI): 1.81 (1.37–2.38)]. Severe knee pain was an independent predictor for falls after adjustment for functional impairment and psychological status. Mild, moderate, and severe knee pain had a specific indirect effect on falls through reducing functional impairment, which in turn increases their psychological concern.

**Conclusion:** Future studies should explore this relationship prospectively and evaluate whether interventions which alleviate psychological concerns and improve function will reduce falls risk in those with mild to moderate knee pain.

## Introduction

As the global population is aging rapidly, falls have become an increasingly prominent public health concern (1). Approximately one in three older persons report at least one fall over the previous 12 months (2). Complications resulting from falls may include severe life-threatening injuries such as subdural hematoma, hip fractures and spinal cord injuries (2). Hospitalization and treatment costs associated with falls contribute substantially to healthcare costs and has been increasing (3, 4). Current cost estimates usually do not include social and psychological costs, which include increased dependency, institutionalization, fear of falling, and quality of life (QoL) (5). Falls are multifactorial events, common risk factors for falls include older age, female gender, previous falls, gait and balance problem, poor vision, and other chronic diseases (6).

Osteoarthritis is a major cause of disability world-wide, with the knee being the commonest affected joint (7). Published literature on the association between falls and knee osteoarthritis has been conflicting, with the lack of an agreed operational definition for osteoarthritis potentially impeding progressing in this area (8). Clinically agreed definitions, which include both radiographic and clinical assessments, are not practically in large community-based studies. Community-based studies have therefore predominantly adopted the symptom of knee pain as a surrogate marker of the presence of knee OA in the community (9–11).

Evidence-based strategies for falls prevention include exercise, home hazards intervention, first cataract surgery and multifactorial interventions (12). These intervention studies for fall prevention have predominantly been conducted among general older populations within the community, institutions or hospitals. Few studies have determined modifiable risk factors in specific conditions such as OA. This study was conducted to determine the relationship between knee pain and falls among community-dwelling older persons, and to further evaluate the role of functional performance and psychological symptoms in this potential relationship.

## Materials and Methods

### Study Design and Population

This was a baseline analysis of Malaysian Elders Longitudinal Research (MELoR) study. Data collected at baseline was utilized in this study. The MELoR study is a longitudinal cohort study based in Kuala Lumpur and its surrounding suburbs (Klang Valley) (13). Individuals aged 55 years and above were selected through simple random sampling stratified by age deciles and ethnicity. The electoral rolls of the Parliamentary constituencies of Petaling Jaya North, Petaling Jaya South, and Lembah Pantai were used as the sampling frame. Data was collected between November 2013 to October 2015. This study was approved by the University of Malaya Medical Center Medical Ethics Committee (Ref: 925.4) and complied with the Helsinki Declaration of 1975, revised in 1983. Written informed consent was obtained from all study participants prior to their inclusion. The inclusion criteria were age 55 years and above and able to provide informed consent. Institutionalized older adults and those with communication difficulties, including cognitive impairment, affecting their ability to respond to the questionnaire were excluded.

### Data Collection

Participants were visited at their own homes initially to recruit them into the study. A structured interview using a computer aided questionnaire was completed during this encounter. Participants were then requested to attend a hospital-based health check clinic. Anthropometric measurements including height, weight, waist, and hip circumference were collected during the hospital visit. The presence of co-morbidities including hypertension, diabetes mellitus (DM), stroke, heart disease, osteoarthritis, and Parkinson's disease was identified by asking participants whether they had ever been told by a doctor about their condition. Presence of urinary continence was identified by enquiring about urine leakage when they coughed or before they got to the toilet. The occurrence of falls was determined by asking participants during their home-based interviews whether they had at least one fall in the past 12 months.

#### Case Definition

To establish the presence of knee pain and osteoarthritis symptoms, participants were asked to indicate whether are often troubled with pain, and to then indicate the parts of their body they experienced pain. Those who indicated they had knee pain were then asked to identify whether this occurred in the left, right, or both knees and to rate the severity of pain either as mild, moderate, or severe.

### Functional Performance

Functional performance in our participants was determined by the *Katz Index of Independence in Activities of Daily Living* (ADL), items from *the Lawton-Brody Instrumental Activities of Daily Living* (IADL), and the Timed Up and Go test.

The Katz ADL tool assessed seven basic ADLs, including walking, bathing, grooming, dressing, eating, transferring, and using the toilet (14). Each item would be assigned the score of “1” if the participant was able to perform that particular activity independently, and “0” if they required any form of supervision or assistance. The maximal score was therefore “7,” which implied the participant was independent of all basic ADLs.

Seven out of eight items of the Lawton-Brody IADL were included in the home-based interview questions (ability to use telephone, going out, shopping, food preparation, doing housework, taking own medication, and ability to handle finances). The item on managing own laundry was removed as the expert panel felt this overlapped with housework. Participants were scored “1” if they “answered phone calls,” “used transportation with assistance,” “shopped independently,” “prepared meals independently,” “did housework with help,” “managed their own medications” and “managed everyday finances with help with banking and major transactions,” or “0” if they function below the above stipulated levels for each item (15). The maximal score was therefore “7.”

*The Timed Up and Go test* was first demonstrated to the participant, followed by one trial run, before taking a second measurement which was recorded. Shoes were kept on for this test. The time taken for the participant to complete a 3-m continuous walk from and back to a seated position on a standardized chair, 46 cm in height, with arms and a back rest was recorded. Participants were instructed to walk independently at their natural pace and were allowed to use a walking aid if they normally required one. Completion time of longer than 13.5 s (s) indicated impaired lower limb function (16).

### Psychological Concern

Depression, anxiety and stress were measured with the *21-item Depression, Anxiety and Stress Scale (DASS-21)*. This was a self-reported measure in which participants rated the frequency and severity of the negative emotions of depression (e.g., loss of self-esteem/incentives and depressed mood), anxiety (e.g., fear and anticipation of negative events), and stress (e.g., persistent state of over arousal and low frustration tolerance) over the previous week. Frequency or severity ratings are made on a 4-point Likert scale, with 0 indicating “did not apply to me at all” and 3 indicating “applied to me very much, or most of the time.” The scores were calculated individually for the three components: depression, anxiety and stress (17). In addition, participants were asked whether they were afraid of falling. Those who answered “yes” to this question were considered to have fear of falling (18).

### Patient and Public Involvement Statement

As this was a population-based cohort study, non-governmental organizations, senior citizens' groups and charitable organizations were consulted in a consultation forum followed by focus groups to identify key issues they felt they would like addressed. The MELoR questionnaire was scrutinized by older persons from local senior citizens' groups and their feedback given due consideration in refining the questionnaire. Our recruitment process involved engaging local community leaders who then assisted us with first organizing local publicity events which included health talks, free health screening, or exercise sessions. Selected participants were then accessed by identifying these individuals or their neighbors at these events, and enlisting the help of local residents in door-to-door recruitment. Participants were provided with individual feedback during their health check by a medical specialist and this was followed by a written report of their screening results. Preliminary aggregated results have been presented to local residents through numerous local follow-up publicity events, and further individual feedback is planned during subsequent waves.

### Statistical Analysis

Data analyses were conducted using SPSS Version 20 (IBM, Armonk, NY, USA). Descriptive statistics were first presented in mean with standard deviation for continuous data and frequencies with percentages for categorical data. The independent *t*-test was applied for continuous variables and chi-squared tests were performed for nominal variables in bivariate analysis. Subsequently, the odds ratios (OR) with 95% confidence intervals (CI) were determined for risk of having fallen in the preceding 12 months, separately for participants with and without knee pain. In addition, similar comparisons were made for severity of knee pain using logistic regression with dummy variables. Uncertain responses and missing values were removed in the association analysis. Multiple logistic regressions analyses were performed to assess the association between knee pain as well as knee pain severity and falls following adjustments for potential confounders and mediators. Potential confounders were selected based on differences in baseline characteristics and clinical relevance.

**Figure 1:**
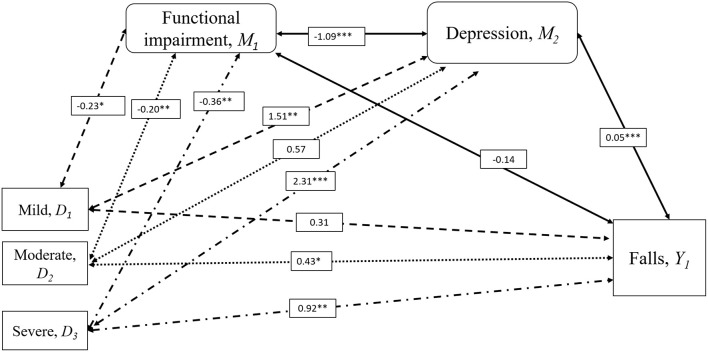
Mediation Analysis for the Influence of Depression on Falls and Knee Pain Severity. Serial multiple mediation analysis with bootstrapping compared to severity of knee pain with falls risk adjusted for age, sex, diabetes, incontinence, and visual impairment. Moderate (D_2_) and severe knee pain (D_3_) was associated with a history of falls in the past 12 months (Y_1_), but not mild knee pain (D_1_) compared to the individuals with no knee pain. Increased risk of falls in moderate and severe knee pain were mediated by depression (M_2_) resulting from functional impairment (M_1_). Significance indicated by *p < 0.05, **p < 0.01, ***p < 0.001. Numbers were standardized β coefficients. IADL scores were used to represent functional impairment.

**Figure 2:**
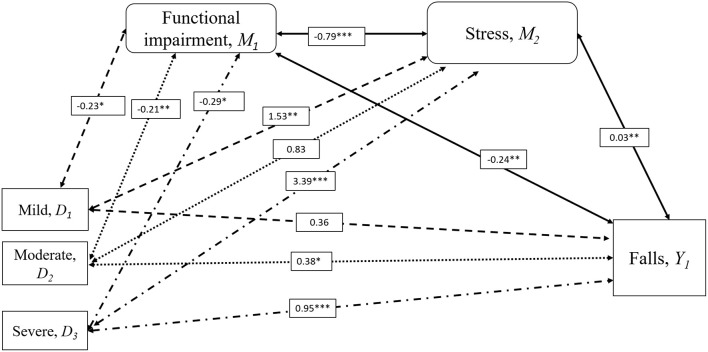
Mediation Analysis for the Influence of Stress on Falls and Knee Pain Severity. Serial multiple mediation analysis with bootstrapping compared to severity of knee pain with falls risk adjusted for age, sex, diabetes, incontinence, and visual impairment. Moderate (D_2_) and severe knee pain (D_3_) was associated with a history of falls in the past 12 months (Y_1_), but not mild knee pain (D_1_) compared to the individuals with no knee pain. Increased risk of falls in moderate and severe knee pain were mediated by stress (M_2_) resulting from functional impairment (M_1_). Significance indicated by *p < 0.05, **p < 0.01, ***p < 0.001. Numbers were standardized β coefficients. IADL scores were used to represent functional impairment.

Serial multiple mediation analysis was then applied to evaluate the role of depression, stress and IADL scores in the association between knee pain severity and falls (see Figures 1, 2). These mediators were selected based on the condition of a significant association with falls controlled with demographic differences (**Table 3**) and were assumed to have a direct effect on each other. The independent variable (severity of knee pain) is assumed to influence mediators in a serial way that ultimately influences the dependent variable (falls). The mediation model was tested using the SPSS macro PROCESS. This allows the estimation of path coefficients for more than one mediator and the generation of 95% bias-corrected (BC) bootstrap CI based on 10,000 bootstrap samples. A mediation effect is confirmed when knee pain severity, presented using dummy variables (D_1−3_), affects a dependent outcome variable Y (falls) through one or more potential intervening variables or mediators (M) (19). The respective indirect effect was estimated by its standard error as reported in **Table 4** (20). It indicated the extent to which the dependent variable (falls) changes when the independent variable (knee pain severity) is held fixed and the mediator variable (functional impairment, depression, or stress) changes by the amount it would have changed had the independent variable increased by one unit (21).

## Results

### Study Population

Data on knee pain, falls and physical function were available in 1,212 participants. Four hundred and two participants (33.17%) reported the presence of knee pain, of who 124 (30.85%) had mild, 210 (52.24%) had moderate, and 68 (16.92%) had severe knee pain.

### Basic Characteristics of Subjects With and Without Knee Pain

Characteristics associated with knee pain included being female, unmarried or divorced, low educational level, increased BMI, presence of diabetes, hypertension incontinence, osteoarthritis, and polypharmacy. Participants with knee pain had poorer functional performance and psychological status (Table 1).

**Table 1:** Baseline characteristics of participants.

	***N***	**No knee pain** **(*n* = 810, 66.8%)**	**Knee pain** **(*n* = 402, 33.2%)**	***p*-value**
Age, years, mean (SD)	1,212	68.84 (7.43)	69.08 (7.48)	0.639
Gender, female n (%)	1,212	421 (52.0)	267 (66.4)	*<0.001*
Unmarried/divorced	1,210	204 (25.2)	127 (31.7)	*0.018*
Primary school education or lower	1,211	189 (23.4)	148 (36.8)	*<0.001*
BMI, kg/m^2^, mean (SD)	1,027	24.71 (4.30)	26.71 (5.08)	*<0.001*
Comorbidities, n (%)	1,212			
Diabetes mellitus		225 (27.8)	150 (37.3)	*0.001*
Heart disease		99 (12.2)	45 (11.2)	0.602
Hypertension		398 (49.1)	249 (61.9)	*<0.001*
Stroke		13 (1.6)	7 (1.7)	0.861
Visual impairment		315 (38.9)	173 (43.0)	0.166
Incontinence		161 (19.9)	114 (28.4)	0.001
Osteoarthritis		44 (5.4)	64 (15.9)	*<0.001*
Parkinsonism		5 (0.6)	1 (0.2)	0.389
Functional performance				
Total katz, mean (SD)	1,212	5.11 (0.67)	5.16 (0.76)	0.250
Total lawton, mean (SD)	1,212	6.59 (0.92)	6.32 (1.13)	*<0.001*
TUG score, mean (SD)	1,009	11.98 (3.24)	13.77 (5.74)	*<0.001*
Psychological status,				
Depression, mean (SD)	1,193	2.52 (4.48)	4.10 (6.38)	*<0.001*
Anxiety, mean (SD)	1,184	3.19 (3.84)	4.91 (4.99)	*<0.001*
Stress, mean (SD)	1,190	3.84 (5.11)	5.61 (7.30)	*<0.001*
Fear of falling (yes/no)	1,205	566 (70.0)	345 (86.9)	*<0.001*
Polypharmacy (>5 medications)	1,159	262 (32.8)	174 (45.7)	*<0.001*

*Italicized fonts indicate significance at p < 0.05; SD, standard deviation; BMI, Body Mass Index; TUG, Timed Up and Go*.

### Factors Associated With Falls According to Knee Pain Status

Table 2 summarizes the odds ratio for falls by knee pain and each covariate in those with and without knee pain followed by sub-group analysis according to knee pain severity. Presence of knee pain showed significant association with increased in falls risk (OR = 1.81; 95%CI = 1.37–2.38). All three stages of knee pain severity were significantly associated with increased risk of falls compared with no knee pain (Mild: OR = 1.57; 95%CI = 1.02–2.42, Moderate: OR = 1.55; 95%CI = 1.11–2.25, Severe: OR = 2.92; 95%CI = 1.72–4.97), respectively.

**Table 2:** Risk of falls according to knee pain or knee osteoarthritis status.

	Falls OR (95% CI)*				
	**Knee pain**		**Knee pain severity**		
	**Absent**	**Present**	**Mild**	**Moderate**	**Severe**
Odds ratio for falls No Knee Pain (reference)	1	*1.81 (1.37–2.38)*	*1.57 (1.02–2.42)*	*1.55 (1.11–2.25)*	*2.92 (1.72–4.97)*
**Covariates**					
Age (years)**	*1.05 (1.02–1.07)*	1.02 (0.9–1.05)	1.03 (0.97–1.08)	1.01 (0.97–1.05)	1.04 (0.98–1.11)
Gender, female	1.31 (0.92–1.86)	1.24 (0.78–1.96)	1.26 (0.54–2.97)	1.61 (0.83–3.12)	0.50 (0.17–1.47)
Unmarried/divorced	*1.63 (1.12–2.38)*	1.26 (0.81–1.98)	1.57 (0.67–3.66)	1.04 (0.54–2.01)	1.19 (0.45–3.15)
Primary school or lower	1.36 (0.92–2.03)	1.23 (0.79–1.90)	1.18 (0.53–2.63)	1.44 (0.77–2.69)	0.90 (0.34–2.44)
BMI (kg/m^2^)**	*1.05 (1.01–1.10)*	0.98 (0.93–1.02)	*0.89 (0.80–0.99)*	0.95 (0.88–1.02)	1.04 (0.96–1.14)
**Covariates**					
Diabetes mellitus	*1.53 (1.06–2.22)*	1.19 (0.77–1.84)	1.63 (0.72–3.70)	1.17 (0.63–2.18)	0.67 (0.25–1.76)
Heart disease	0.99 (0.58–1.68)	0.82 (0.41–1.64)	0.61 (0.12–3.03)	0.47 (0.13–1.67)	0.86 (0.25–3.01)
Hypertension	1.13 (0.80–1.60)	0.80 (0.52–1.23)	0.91 (0.41–2.03)	0.78 (0.42–1.49)	0.51 (0.18–1.44)
Stroke	1.25 (0.34–4.61)	1.74 (0.38–7.89)	1.33 (0.12–15.20)	2.71 (0.17–44.13)	1.20 (0.09–20.01)
Visual Impairment	*1.57 (1.11–2.24)*	1.50 (0.98–2.30)	1.61 (0.73–3.56)	1.77 (0.96–3.28)	0.71 (0.27–1.86)
Incontinence	1.44 (0.95–2.17)	*1.79 (1.13–2.82)*	1.39 (0.59–3.29)	1.69 (0.87–3.29)	2.22 (0.82–6.00)
Functional performance**					
Total katz	0.82 (0.65–1.03)	1.16 (0.86–1.57)	0.93 (0.57–1.50)	*1.98 (1.07–3.64)*	0.94 (0.60–1.51)
Total lawton	*0.69 (0.58–0.81)*	*0.82 (069–0.99)*	0.74 (0.54–1.02)	0.98 (0.73–1.33)	0.75 (0.50–1.12)
TUG score, s	*1.12 (1.06–1.18)*	1.01 (0.97–1.05)	1.01 (0.95–1.06)	1.00 (0.92–1.09)	1.02 (0.93–1.12)
Psychological status**					
Depression	*1.06 (1.02–1.09)*	*1.06 (1.02–1.09)*	1.03 (0.98–1.09)	*1.12 (1.05–1.19)*	1.01 (0.95–1.08)
Anxiety	*1.05 (1.00–1.09)*	1.04 (0.99–1.08)	1.06 (0.98–1.15)	1.02 (0.95–1.09)	1.02 (0.94–1.10)
Stress	1.02 (0.99–1.06)	*1.05 (1.02–1.08)*	1.03 (0.98–1.09)	*1.08 (1.03–1.13)*	1.02 (0.97–1.07)
Fear of falling	1.15 (0.78–1.70)	1.37 (0.70–2.67)	1.26 (0.42–3.79)	2.60 (0.74–9.16)	0.86 (0.21–3.56)
Polypharmacy (Medication>5)	1.07 (0.74–1.55)	1.31 (0.84–2.04)	1.45 (0.64–3.31)	1.07 (0.57–2.01)	1.34 (0.45–3.96)

Italicized fonts indicate significance at p < 0.05* occurrence of at least one fall in the preceding 12 months. OR, Odds ratio; SD, standard deviation; BMI, body mass index; TUG, Timed Up and Go; s, seconds. * ≥ 1 fall in past 12 months** per unit increase, the OR of falls risk of each covariate was analyzed according to presence of knee pain and status of knee pain severity.

Older age, being unmarried, or divorced, increased in BMI, having diabetes and visual impairment, poor IADL (Lawton) and poor TUG score, and increased depression and anxiety scores were significantly associated with falls in those without knee pain. In individuals with knee pain, urinary incontinence, poorer IADL score, increased in depression scores, and stress were significant associated factors with falls.

Individuals with knee pain were then evaluated according to knee pain severity. In those with mild knee pain, increased BMI was significantly associated with increased risk of falls. While in individuals with moderate knee pain, better IADL scores appeared to increase their risk of falling. Increased of depression and stress scores were significantly associated with falls. In individuals with severe pain, no significant associated factors were identified (Table 2).

### Multivariate Analysis on Presence and Severity of Knee Pain and Falls

Table 3 summarizes the results of the multivariate analyses to examine the association between both presence of knee pain and severity of knee pain following multiple adjustments for potential confounders and mediators. The presence of knee pain was associated with an increased risk of falls after adjustment for potential confounders (OR = 1.65; 95%CI = 1.25–2.19). This relationship remained unchanged after separate adjustments for both functional impairment represented by IADL scores and Timed Up and Go scores. Following adjustment for age, sex, diabetes, incontinence and visual impairment, however, there was no longer a significant association between falls and mild knee pain (OR = 1.46; 95%CI = 0.94–2.27), while moderate (OR = 1.49; 95%CI = 1.04–2.13) and severe knee pain (OR = 2.62; 95%CI = 1.52–4.52) remained significantly associated with falls compared with no knee pain. The relationship between moderate knee pain and falls was attenuated by additional adjustment for IADL scores (OR = 1.42; 95%CI = 0.97–2.04), but not with adjustment for Timed Up and Go, depression or stress scores. The relationship between severe knee pain and falls was unattenuated by any further adjustments for functional impairment scores or psychological scores.

**Table 3:** Multivariate analysis on the influence of knee pain and knee pain severity on falls.

	**Falls, odds ratio (95% CI)**				
	**Adjustment 1**	**Adjustment 2 (a)**	**Adjustment 2 (b)**	**Adjustment 3 (a)**	**Adjustment 3 (b)**
**Knee pain**, ***n*** **(%)**					
Absent	1.00 (ref)	1.00 (ref)	1.00 (ref)	1.00 (ref)	1.00 (ref)
Present	*1.65 (1.25–2.19)*	*1.57 (1.18–2.09)*	*1.71 (1.25–2.35)*	*1.61 (1.20–2.14)*	*1.61 (1.20–2.15)*
No of participants in model	1,208	1,208	1,009	1,193	1,190
**Knee pain severity**					
No Pain	1.00 (ref)	1.00 (ref)	1.00 (ref)	1.00 (ref)	1.00 (ref)
Mild Pain	1.39 (0.90–2.16)	1.32 (0.89–2.06)	1.38 (0.85–2.26)	1.28 (0.82–2.02)	1.41 (0.90–2.22)
Moderate Pain	*1.47 (1.03–2.10)*	1.40 (0.98–2.01)	*1.57 (1.05–2.34)*	*1.49 (1.04–2.15)*	*1.44 (1.00–2.07)*
Severe Pain	*3.05 (1.81–5.15)*	*2.83 (1.67–4.81)*	*3.12 (1.75–5.55)*	*2.90 (1.69–4.97)*	*2.99 (1.74–5.14)*
No of participants in model	1,208	1,208	1,009	1,195	1,190

Italicized fonts indicate significance at p < 0.05.

Adjustment 1: age, sex, and comorbidities (diabetes, incontinence and visual impairment),.

Adjustment 2: Adjustment 1 + functional impairment [(a) IADL Lawton, (b) Timed Up and Go],.

*Adjustment 3: Adjustment 1 + psychological status [(a) Depression and (b) Stress]*.

### Serial Multiple Mediation Analysis on the Relationship Between Knee Pain Severity and Falls

Figures 1, 2 and Table 4 demonstrate the results of the serial multiple mediations analyses evaluating the mediation effects of functional impairment measured with IADL scores and depression (Figure 1) and stress (Figure 2). The absence of knee pain was considered as the reference variable. The values presented in the figures represent the standardized β coefficients, while relationships with were statistically significant to the level of *p* < 0.05 were accompanied by a single asterisk, *p* < 0.01 a double asterisk, and *p* < 0.001 a triple asterisk.

**Table 4:** Specific indirect effects of mediators for the knee pain severity and falls.

	**Indirect effect, Effect, Boostrap SE, (95% LLCI, ULCI)**		
	**Indirect effect 1**	**Indirect effect 2**	**Indirect effect 3**
**Model 1 (*****N*** **=** **1,180)**	**Severity↔functional impairment↔Falls**	**Severity↔functional impairment** **↔Depression↔Falls**	**Severity↔Depression↔Falls**
Mild	0.032, 0.026 (−0.003–0.097)	*0.012, 0.007 (0.001–0.028)*	*0.069, 0.036 (0.010–0.151)*
Moderate	0.028, 0.018 (−0.002–0.068)	*0.010,0.005 (0.002–0.023)*	0.026, 0.023 (−0.009–0.081)
Severe	0.050, 0.035 (−0.004–0.131)	*0.018, 0.013 (0.002–0.052)*	*0.106, 0.053 (0.022–0.230)*
**Model 2 (N=1175)**	**Severity↔functional impairment↔Falls**	**Severity↔functional impairment** **↔Stress↔Falls**	**Severity↔Stress↔Falls**
Mild	*0.042, 0.029 (0.001–0.112)*	*0.005, 0.004 (0.000–0.015)*	*0.044, 0.027 (0.002–0.106)*
Moderate	*0.039, 0.020 (0.006–0.083)*	*0.005, 0.003 (0.004–0.013)*	0.024, 0.019 (−0.003–0.070)
Severe	*0.054, 0.036 (0.003–0.140)*	*0.007, 0.006 (0.0001–0.0240)*	*0.097, 0.0512 (0.012–0.210)*

Italicized fonts indicate significance at p < 0.05.

SE, Standard error; LLCI, Lower Limit Confidence Interval; ULCI, Upper limit confidence interval.

*Both models were adjusted for age, sex, marital status, education level, diabetes, incontinence, and visual impairment*.

Figure 1 illustrates the mediation effects of functional impairment and depression on the bidirectional relationship between knee pain severity and falls. In this analysis, moderate and severe knee pain were more likely than no knee pain to be associated with falls, but not mild knee pain. Functional impairment did not directly affect falls risk in this analysis. However, an indirect effect was observed between mild, moderate and severe knee pain with increasing functional impairment, identified through reducing IADL scores, with increasing symptoms of depression, which in turn is associated with an increased risk of falls (D_1_,D_2_,D_3_ ↔M_1_↔M_2_↔Y_1_) (Table 4). The indirect effect of depression on falls in knee pain was also examined, with there being a presence of a significant effect in mild knee pain and severe knee pain compared to no knee pain, but not moderate knee pain (D_1_,D_3_ ↔M_2_↔Y_1_). This suggests that depression demonstrates a mediating effect on the relationship between mild and severe knee pain and falls. While functional impairment, is related to all three severity of knee pain, it is not related to an increased risk of falls unless there is presence of associated depression.

Figure 2 illustrates the roles of functional impairment and stress in determining the association between knee pain severity and falls. With no knee pain as the reference variable, moderate and severe knee pain but not mild knee pain was associated with falls after adjustment for potential confounders. The first indirect effect was between functional impairment and falls, whereby all three knee pain severities were associated with functional impairment, and functional impairment is in turn associated with falls (D_1_,D_2_,D_3_ ↔M_1_↔Y_1_). The second indirect effect was the serial effect of knee pain severity on functional impairment and functional impairment on stress and stress on falls (D_1_,D_2_,D_3_ ↔M_1_↔M_2_↔Y_1_). The third indirect effect was observed between mild and severe knee pain with stress, which in turn was associated with falls (D_1_, D_3_ ↔M_2_↔Y_1_) (Table 4). The relationship between mild, moderate, and severe knee pain with falls was therefore mediated by functional impairment which is directly related to falls as well as indirectly through stress, while the association between mild and severe knee pain and falls was mediated by stress.

## Discussion

The presence of knee pain is linked to fall occurrence in the previous 12 months. When the severity of knee pain was considered, mild knee pain was no longer associated with increased risk of falls compared to those with no knee pain once adjusted for potential confounders. The relationship between moderate knee pain and falls was accounted for by functional impairment measured with the Lawton IADL scale. The increased risk of falls in those with severe knee pain above those with no knee pain was accounted for by psychological or functional deficits. Additional mediation analyses suggest that the functional impairment associated with mild, moderate and severe knee pain was not independent from the influence of depression. Both symptoms of depression and stress identified with DASS-21 however explained the indirect influence of functional impairment on falls in participants with mild, moderate and severe knee pain and also directly influenced the relationship between mild and severe knee pain and falls.

Our findings were consistent with that of previous studies in suggesting a positive relationship between knee pain and falls (11, 22–25). The presence of knee pain was associated with an 81% increased odds of any fall in the preceding 12 months in our population. The presence of knee pain in any older population is likely to be predominantly determined by the presence of osteoarthritis. In a previous study conducted in a primary care setting, radiographic diagnosis of osteoarthritis were present in 53% individuals aged 55 years who reported the presence of knee pain during a postal survey (26). As this study was cross-sectional in nature, the relationship between knee pain and falls should be considered as bidirectional. It is plausible that those with severe knee pain or depression would recall their falls more clearly than those who do not. Nevertheless, the handful of studies which have examined risk factors for falls in those with knee pain or osteoarthritis have only assessed muscular strength and balance. Our study has therefore added to existing knowledge by assessing the influence of functional and psychological status in this relationship.

A previous study had suggested that individuals with mild lower limb osteoarthritis on radiological examination were significantly less likely to fall than those with no radiological evidence of osteoarthritis (8). The study population in the previous study comprised of those with recurrent and injurious falls rather while this present study assessed the presence of any fall in the previous 12 months. Furthermore, radiological changes of osteoarthritis are known to correlate poorly with symptoms (22). The results of the two studies were, however, similar in the level of association between severe pain and falls, in that individuals with severe knee pain had twice the odds of falling compared with subjects without symptoms.

While the presence of knee pain is likely to adversely affect physical performance, our study found that this did not appear to influence the relationship between knee pain and falls regardless of severity of pain (27). The relationship between moderate knee pain and falls was attenuated by functional impairment rather than physical performance or psychological status. The relationship between severe knee pain and falls was, however, independent of functional, or psychological scores.

Our mediation analyses suggest that stress and depression mediated the bidirectional relationship between mild and severe knee pain with falls. Moderate knee pain appeared not be related to stress or depression. Pain coping management in addition to weight management reduces psychological disability in patients with knee OA (28). It is plausible that those with moderate pain may have developed coping mechanisms for pain while those with mild symptoms who are at an earlier stage of OA and are still learning to cope. The presence of severe pain, however, then compromises the ability to cope. The relationship between depression, falls and pain had been demonstrated in a cross-sectional analysis of a large clinical dataset of older individuals assessed for home care (29). However, this study had enquired about general pain rather than knee pain specifically (29). Other studies have suggested that depression and chronic pain may have their own independent pathway to falls (30) and both pain (31) and depression (32) may interfere with the older adults' cognition and executive function consequently resulting in falls. It is also possible that anti-depressants used in those who are depressed are implicated in their falls (32). Fear of falling on the other hand did not appear to influenced the knee pain-falls association. We had established the presence of fear of falling using a single question which has been found to be as reliable as using a falls efficacy questionnaire. Furthermore, a separate study has also established the lack of association between fear of falling and falls (18, 33, 34).

In this population-based study, self-reported symptoms of knee pain was not further evaluated to confirm the presence of knee osteoarthritis either clinically or radiologically, as exposure to ionizing radiation could not be justified and clinical expert assessments were not feasible in large population-based studies such as ours. An undefined proportion of our participants would therefore suffer from knee pain from other conditions affecting the knee joint or from injuries affecting the soft tissue around the knee which may even have resulted from a fall. Other joint conditions are, however, known to be less common than osteoarthritis in older individuals (35). The self-reported falls history also introduces the possibility of recall bias which cannot be avoided involve of the cross-sectional design. Our study will therefore need to be complemented by prospective follow-up studies evaluating the longitudinal relationship between osteoarthritis of the knee and falls. Information on orthostatic hypotension, medications, and environmental hazards and falls have been collected in detail in the MELoR study, however, due to the complexity of the relationship between these three major risk factors and falls particularly in those with osteoarthritis, we have not included these in factors this study. Further studies should therefore evaluate the relationship between these three major risk factors and falls in individuals with osteoarthritis in greater detail.

The presence of knee pain is associated with increased risk of falls in the previous 12 months, with severe knee pain remaining as independent associated factors after adjustment for potential confounders. Both functional and psychological status mediate the association between moderate and severe knee pain and falls. Studies on falls prevention specifically targeting individuals with knee pain should incorporate interventions which improve functional and psychological outcomes.

## Data Availability Statement

Due to concerns about loss of fidelity of personally identifiable data, the MELoR data set is currently not available publicly. However, parts of the data set will be released anonymised through written requests submitted to the corresponding author.

## Ethics Statement

The studies involving human participants were reviewed and approved by University of Malaya Medical Center Medical Ethics Committee (Ref: 925.4). The patients/participants provided their written informed consent to participate in this study.

## Author Contributions

A-VC, NH, SO, SK, and MT conceived the study, contributed to study design, obtained the funding for the study and were responsible for the conduct of the study. SM was involved in data collection. SM and MT contributed to data analysis. All authors contributed toward the writing of the manuscript and approved the final submitted version.

### Conflict of Interest

The authors declare that the research was conducted in the absence of any commercial or financial relationships that could be construed as a potential conflict of interest.
